# Comparison of two dimensional and live three dimensional ultrasounds for the diagnosis of septated uterus

**Published:** 2014-08

**Authors:** Maryam Niknejadi, Farnaz Akhbari, Fatemeh Niknejad, Gholamreza Khalili, Marzieh Shiva

**Affiliations:** 1 *Department of Reproductive Imaging at Reproductive Biomedicine Research Center, Royan Institute for Reproductive Biomedicine, ACECR, Tehran, Iran.*; 2 *Department of Epidemiology and Reproductive Health at Reproductive Epidemiology Research Center, Royan Institute for Reproductive Biomedicine, ACECR, Tehran, Iran.*; 3 *Department of Endocrinology and Female Infertility at Reproductive Biomedicine Research Center, Royan Institute for Reproductive Biomedicine, ACECR, Tehran, Iran.*

**Keywords:** *Congenitalabnormalities*, *Ultrasonography*, *hysteroscopy*

## Abstract

**Background:** Traditionally, septate uterus was diagnosed with invasive method like hysterosalpingography and hysteroscopy. Nowadays transvaginal ultrasonography was reported to be a sensitive tool for detection of septate uterus too.

**Objective:** The objective of the present study was to evaluate the application of two dimensional ultrasound (2-DUS) and real time three dimensional ultrasound (3-DUS) in differentiating various type of septated uterus. Hysteroscopy confirmation was assigned as the gold standard.

**Materials and Methods: **This retrospective study was performed among 215 infertile women with suspected septate uterus from October 2008 to July 2012. An inclusion criterion was septated uterus based on HSG or experiencing abortion, preterm labor, or recurrent IVF failure. Fusion anomalies were excluded from the study (unicornuate, bicornuate and didelphys anomalies). The results of 3D and 2D sonographies were compared, while they were confirmed by hysteroscopy result in detection of septated uterus. Kappa index for agreement between 2DUS and hysteroscopy, as well as 3-DUS and hysteroscopy in detection of septate uterus was carried out. By receiver operating characteristic (ROC) curve, cut off points for predicting the kind of anomalies were proposed.

**Results: **The women were evaluated by 2-DUS (n=89) and (II) 3-DUS (n=126). All women underwent hysteroscopy, following 2-DUS and 3-DUS at the same or subsequent cycle. The results of kappa (K) index were 0.575 and 0.291 for 3-DUS and hysteroscopy, as well as 2-DUS and hysteroscopy, respectively. Also, the cutoff points were 27% for arcuate and subseptate, and 35% for differentiating septate and subseptate.

**Conclusion:** Real time 3-DUS has better ability for visualization both uterine cavity and the fundal uterine, so it has higher agreement in detection of septate uterus than 2-DUS.

## Introduction

Less than half of the population with congenital mullerian disease (CMD) manifest clinical symptoms and most of them denoted to septate uterus (close to 50%) compared with other malformations (1). Traditionally, septate uterus was diagnosed by x-ray hysterosalpingography or diagnostic hysteroscopy. More recently transvaginal ultrasonography was reported to be a sensitive tool for detection of uterine anomalies (2, 3). 3DUS is minimally invasive approach permits the obtaining of anatomic images of endometrium and myometrium, accurate depiction of the septate uterus, and even the measurement of septal height and thickness (4, 5).

Unlike 2-D ultrasound, real time three dimensional ultrasound (3-DUS) can view the coronal surface of the uterus, and it is an important alternative method in the diagnosis of congenital uterine anomalies. Three-dimensional ultrasonography permits the obtaining of planar reformatted sections through the uterus, which allow precise evaluation of the fundal indentation and the length of the septum (6). 

The images obtained in the form of a contiguous series of thin slices [multi-slice (MS) view] or strong multi-resolution images. Scanned volumes are evaluated in multi-planar three-dimension and MS view mode with a slice interval of 0.5-0.6 mm (7). Additionally, laparoscopy and hysteroscopy are considered as invasive methods. So real time 3D US is an important alternative method in the diagnosis of congenital uterine anomalies.

Various studies, conducted in the evaluation of accuracy of 3-DUS in diagnosis of CMA, have compared this method with hysterosalpingography (HSG) and two dimensional ultrasound (2-DUS) or combination of both techniques (8). (Sensitivity, specificity and predictive value for the diagnosis of uterine cavity anomalies were higher for 3-DUS in comparison to 2-DUS or HSG, but it was comparable to combination of both techniques. Comparing live 3-DUS and MRI reveals high degree of concordance between 3-DUS and MRI in the diagnosis of uterine malformation (9). Ghi *et al* depicted extreme accuracy of 3-DUS in diagnosis and classification of CMA (4). 

One study showed that concordance rates among the initial diagnosis by HSG and 3-DUS were 30.4%, 75%, 83% and 80% for bicorne, arcuate, septate and unicorne uterus, respectively. In different cases applied hysteroscopy, the result were 100% in concordance with the 3-DUS evaluations (5). According to these studies, real time 3-DUS is the accurate test for the diagnosis of CMA. We applied both 2-DUS and 3-DUS techniques in detection of CMA and compared their findings with hysteroscopy results. The purpose of this study was to compare agreement of 2-DUS and live 3-DUS in the diagnosis of CMA with respect to the hysteroscopic findings as the gold standard, and to obtain the cut off points for differentiating various type of arcuate, subseptate or septate uterus.

## Materials and methods

This cross sectional retrospective study was conducted at the Reproductive Biomedicine and Imaging Department of the Royan Institute during 46 months period from October 2008 to July 2012. The study was approved by the Royan institute research ethics committee. Written informed consent was obtained from all participants. The study included of 215 patients, 89 women underwent 2-DUS and 126 women underwent 3-DUS. Subsequently, hysteroscopy was performed for 225 patients by expert gynecologists with at least 10 years of experience. The results of hysteroscopy were compared to the findings of US (2D and 3D) to find the agreement. An inclusion criterion was suspected septate uterus based on HSG or after experiencing abortion, preterm labor, or recurrent IVF failure, while patients with fusion anomalies, uterine polyp, fibroid, adhesions or bleeding during ultrasound were excluded.

TVS was performed in follicular phase of the cycle to mid-cycle (from days 5-15 of cycle) using an Aloka α-10 (Medison Co. Japan) with a transvaginal 6-7.5 MHz probe). 3-DUS was done during luteal phase from days 17-21 of cycle. Images from the uterus were obtained with Accuvix XQ (Medison Company, South Korea) machine with transvaginal (TV) 3D probe (5-8MHZ) (Medison Company, South Korea) for real time 3-DUS. For each patient, a hysteroscopy was arranged for the early follicular phase of the same or subsequent cycle. All sonographic examinations were done by an expert radiologist with over 10 years’ experience. The endometrial cavity in 2-DUS was inspected in two perpendicular planes, sagittal and transverse view. Analysis of fundus morphology by 2-DUS was performed in a transverse view, distance between endometrial cornea and the extent of joining this 2 corneal angel is helpful in detecting anomalies.

It depends on mental ability to reconstruct the two-dimensional image into three dimensions in order to make a diagnosis. These images are user dependent that could be acquired by experience. Uterus subdivided to normal, arcuate, subseptate and septate uterus. Specific ultrasound diagnosis of various uterine anomalies was subjective and obtains only by visual imaging experience. The analysis of uterine morphology in (3-DUS) was performed in a standardized reformatted section with the uterus in the coronal view, using the interstitial portions of fallopian tubes as the reference points. Various clinical classified uterine anomalies were presented based on the classification of the American Fertility Society (AFS).

**Figure 1:**
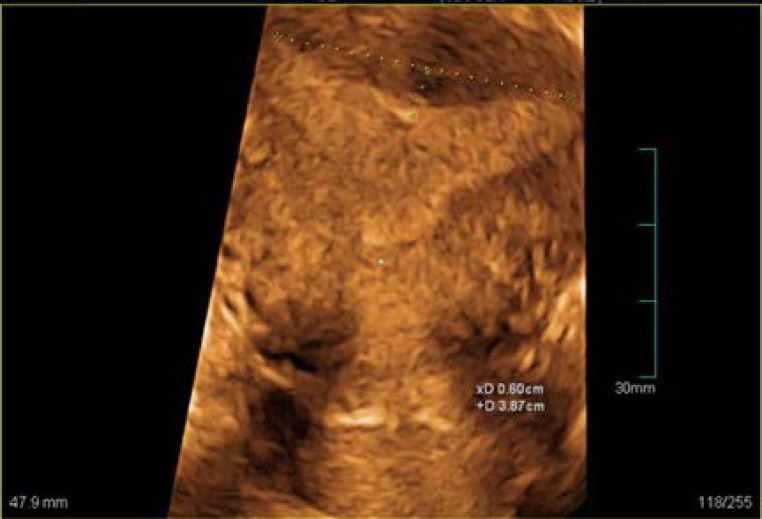
Uterus measuring: in coronal reformatted section, fundal depth is 6 mm and interosteal line is 38.7 mm

Three measurements were taken from patients undergoing 3-DUS as follows: (I) Uterine cavity width was known as the distance between the two internal tubal ostia (referred as interosteal line at the midpoint of uterine cornea), (II) Depth of fundal indentation or the septum length was known as the distance between the midpoint of the line adjoining the tubal ostia and the distal tip of fundal indentation (mm), and (III) Ratio of the septal length to interosteal line (%) (Figure1) (4). Hysteroscopy was used as the gold standard which was performed under general anesthesia using a Storz 4mm hysteroscope (Karl Storz GmbH & Co., Tuttlingen, Germany) by an expert gynecologist.

**Statistical analysis**

Kappa (K) index was applied to assess the agreement between 2D and live 3D ultrasonographies along with hysteroscopy. Receiver operating characteristic (ROC) curve was used, while cut point to differentiate arcuate from short septum and long septum in 3DUS was proposed. Data presented as mean±SD and analysis was carried out using SPSS software (Statistical Package for the Social Sciences, version 16.0), ROC curve, Kappa index and cross tab. 

## Results

In this study, 89 patients experienced 2-DUS, while 126 patients experienced live 3-DUS. Patients in 2-DUS group revealed mean average age of 35.72±5.92 and mean infertility duration of 9.40±5.88. The obtained result revealed various types of infertility among 2-DUS patients as follows: 92.1% with primary infertility, 4.5% with secondary infertility, and 3.4% recurrent abortion. In order to obtain agreement between hysteroscopy with 2-DUS and real time 3-DUS, cross tabs was used (Table I, II). Patient in 3-DUS group has the mean average age of 31.04±5.32 and mean infertility duration of 7.62±5.0. The obtained result also revealed two types of infertility among 3-DUS patients as follows: 96.0% with primary infertility and 4.0% with secondary infertility. Then, cross tab and Kappa index obtained to show Agreement between 3- and 2-DUS and hysteroscopy.

In 3-DUS group, we obtained coronal view of uterus, whereas three lines were major distinction among normal, arcuate, subseptate and septate uterus. In our study, first, septal length and interosteal line were measured, and then the ratio of them was computed. Uterus was known as normal, arcuate, subseptate or septate when the ratio was less than10, 10-20%, 20-35% or more than 35%, respectively. Kappa index was 0.575 for real time 3-DUS and hysteroscopy, indicating a substantial agreement, while this value was 0.291 for 2-DUS and hysteroscopy, indicating a fair agreement. Based on the obtained result of hysteroscopy, we reanalyzed 3-DUS, retrospectively. Average length of in terosteal line and fundal depth in arcuate, subseptate and septate groups, detected by hysteroscopy. These data were depicted in table III. In ROC curve analysis of these results, cut off point between arcuate and subseptate is about 27% with the area of 0.669, whereas cut off point between subseptate and septate is about 35% with the area of 0.777.

**Table I:** Cross tab between the result of 2-DUS and hysteroscopy (as a gold standard)

**2-DUS**	**Hysteroscopy**			
	**Normal**	**Arcuate**	**Subseptate**	**Septate**
Normal	11	4	12	2
Arcuate	5	8	8	1
Subseptate	1	3	18	6
Septate	0	0	3	6
Total	17	15	41	15

Data are presented as number.

**Table II:** Cross tab for the result of 3-DUS and hysteroscopy (as a gold standard)

**3DUs **	**Hysteroscopy**			
	**Normal**	**Arcuate**	**Subseptate**	**Sepatate**
Normal	4	0	0	0
Arcuate	0	2	7	1
Subseptate	1	12	57	3
Septate	0	0	7	31
Total	6	14	71	35

Data are presented as number.

**Table III:** Interosteal line and fundal depth of 3DUS findings based on hysteroscopic classification

	**Length of interosteal line(mm)**	**Fundal depth(mm)**
Arcuate	33.21 ± 4.15	7.75 ± 1.65
Subseptate	32.84 ± 7.31	8.71 ± 2.48
Septate	34.58 ± 9.23	18.18 ± 10.89

Data are presented as mean±SD.

**Table IV:** Sensitivity, specificity, NPV and PPV of detecting CMA by 3DUS

**Study**	**No. of patients**	**Sensitivity**	**Specifity**	**PPV**	**NPV**
Wu et al ( 1997)(9)	40	100	100	100	100
Deutch et al (2008) (8)	13	100	100	100	100

PPV: Positive predictive value NPV: Negative predictive value CMA: congenital mullerian anomaly

Data are presented as %.

**Table V:** Sensitivity, specificity, positive predictive value and negative predictive value in diagnosis of septate uterus by 3DUS

**Evaluations by different studies in applying 3-DUS in detecting MDAs .Study**	**3-DUS objective**	**No. of patients**	**Sensitivity**	**Specificity**	**PPV**	**NPV**
Kupesic & Kurjak (1998)	Detection of a uterine septum	86	98.38	100	100	96
Kupesic et al (2002)	Detection of a uterine septum	894	99.27	100	100	97.61
Jurkovic et al (1998)						
	Detection of a normal uterus	58	98	100	100	94
	Detection of an arcuate uterus	58	100	100	100	100
	Detection of a major uterine anomaly	58	100	100	100	100
Deutch et al (2008)	Detection of a septate uterus	13	100	100	100	100

Data are presented as %.

**Figure 2:**
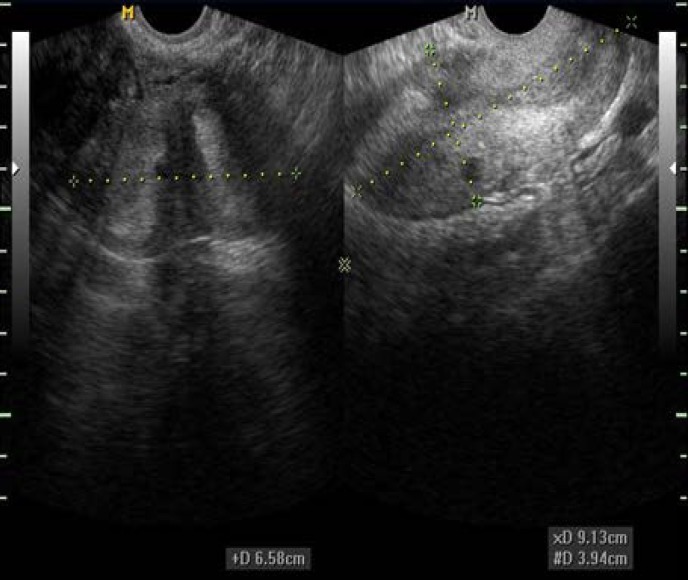
2D Sagittal and transverse views of the septate uterus

**Figure 3:**
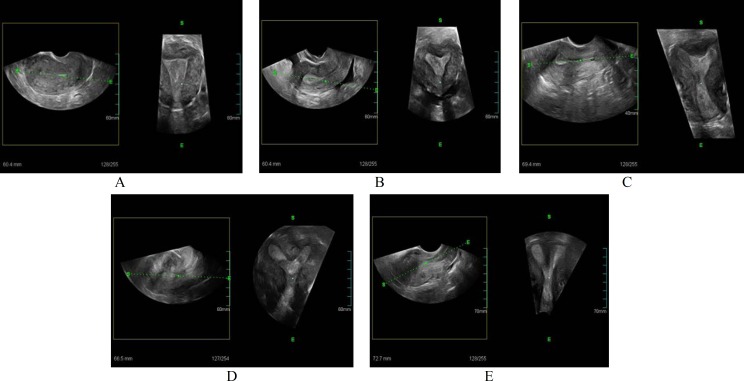
Sagittal and coronal views of the various uterus normal (A), Arcuate (B), Subseptate (C, D), Septate (E).

**Figure 4:**
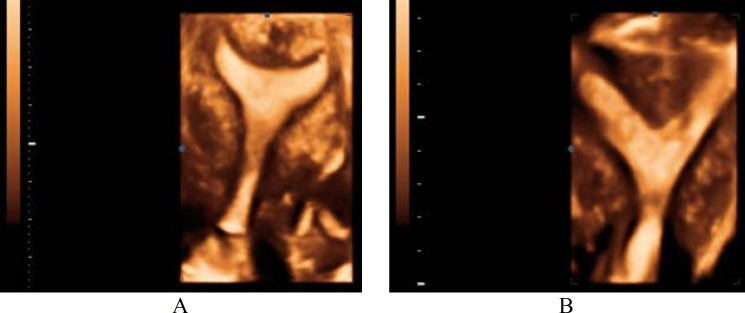
Real time 3D (4D) view of subseptate uterus.

## Discussion

Morphology, thickness and vascularity of the endometrium, as well as the shape of the uterine cavity affect the implantation rate of the embryos (10). The risk of spontaneous miscarriage at the first trimester in patient with septate uterus is 28-45% (10). High prevalence of uterine anomalies among women with recurrent abortion had been documented before (11, 12). 

Accurate diagnosis in patient with recurrent abortion is so important because the types of anomalies determine the treatment. In some cases, surgical treatment may decrease dramatically the risk of recurrent abortion (13). Hysteroscopy is a procedure that cannot be replaced because of therapeutic purpose. Furthermore, it could evaluate the intrauterine cavity which cannot be obtained from ultrasounds. On the other hand, hysteroscopy is the gold standard diagnostic technique for endometrial cavity abnormalities, revealing a suspicious fundal septum which is not recognized in HSG or ultrasound examination (14).

In our study, after ultrasound (3D or 2D), all women were submitted to hysteroscopy, which is considered the most valuable method to detect uterine septum (11). We used hysteroscopy as a gold standard. According to the result of 2DUS in the moini *et al* study in 2013, it is obvious that diagnosis of various septate uterus is not sharply differentiate (15). These findings are confirmed on the basis of the result of our study too, since coronal view is not available through 2DUS (kappa index=0.291). Regarding the fact that Royan institute is a referral infertility center, patient must be checked for uterine anomalies specially septate uterus. 

The result of our previous study in 2012 illustrated various diagnostic indices of TVS compared with hysteroscopy in diagnosing septate and subseptate uterus as follow: sensitivity, specifity, PPV and NPV for subseptate uterus were: 67%, 99.8%, 98.8% and 94.6% respectively. Also sensitivity, specifity, PPV and NPV for septate uterus are 90.0%, 100%, 100%, 98.8% respectively (23). The diagnosis of uterine septum requires the assessment of both internal and external contour of uterine fundus. 

Precise diagnosis is achieved due to the contribution of the C-plan (coronal), while it is impossible to obtain in the majority of cases of 2-DUS, and this is crucial to the diagnosis of these anomalies (Figure 2) (8,12,14). In 3-DUS, planes are stored in order to display a multiplaner view (Figure 3) (16). 3-DUS procedure is less expensive and noninvasive in comparison with hysteroscopy for the assessment of uterine anatomy and diagnosis of mullerian duct abnormalities, so it could be used for further management of this disease (17) (Figure 4). 

Regarding our results, we showed that the diagnostic accuracy of real time 3-DUS was comparable to invasive methods, such as hysteroscopy (Table II). Sensitivity and specificity of 3D in detecting CMA are 100% in the studies by Wu *et al*, Raga *et al*, and Duetch *et al* (9, 18, 19). Also, Momtaz *et al* have showed sensitivity (97%) and specifity (96%) for detection of uterine cavity anomalies. It also showed excellent NPV (99%) and PPV (92%) (8). The results of critical appraisal by Sotirios *et al* have revealed that 3-DUS and hysteroscopy are two of the most accurate diagnostic procedure, while 2DUS is considered as less accurate (Table IV) (20). In studies by Kupesic and Kurjevic, sensitivity, specificity, positive predictive value and negative predictive value in diagnosis of septate uterus by 3-DUS were 98/38.100, 100, and 96%, respectively (21). 

Further study by Kupesic *et al* revealed sensitivity of 99.27%, specificity of 100%, PPV of 100% and NPV of 97.61% that differentiate septate uterus from other types of uterine abnormalities (Table V) (22). In a study by Wu *et al* when they compared 3-DUS and laprascopy and/or hysteroscopy, 3-DUS reveals accuracies of 92% in the diagnosis of septate uterus and 100% for bicorn uterus (9). Regarding these aforementioned studies, the accuracy of real time 3D in detecting CMA is reported and is depicted in tables IV and V. 

Previous studies did not explain about various septate uterus, also they did not calculate cut point between subseptate, septate and arcuate uterus, but we considered various septate uterus and cut point between arcuate, subseptate and septate uterus. Although Moini *et al* found no differentiating between arcuate and short septate uterus, we could differentiate short septum and arcuate uterus by measuring the depth of fundal indentation (23). Our results support other studies about application of 3-DUS as an important method for the assessment and the diagnosis of septate uterus (19, 20, 24). 

In this study, we illustrated ROC curve analysis based on results of hysteroscopic and CMA classification; Cut off point between arcuate and subseptate uteri is about 27%, while cut off point between subseptate and septate uteri is about 35%. This statistics is much closed to the measurement number of our method in this study. On the basis of current study, further prospective studies with large sample size are needed to obtain more accurate cut off point. 

## Conclusion

In conclusion, real time 3D-US is an excellent, noninvasive and accurate technology, which can serve as the gold standard in the assessment of congenital uterine anomalies. Data acquisition time is short and images can be stored for later evaluation, while it can be applied as many times as necessary. 

Therefore, this technique is a valuable problem solving tool, assisting in differentiating between various septate uterus. Further prospective studies in the evaluation of 3-DUS and image reconstruction for diagnosis of septate uterus are needed for confirmation of the agreement between 2-DUS and live 3-DUS in the diagnosis of septate uterus. Also, it is required to consider the classification system based on the ratio of septal length and Interosteal line measurement.
